# Ant Lion Optimization algorithm for kidney exchanges

**DOI:** 10.1371/journal.pone.0196707

**Published:** 2018-05-03

**Authors:** Eslam Hamouda, Sara El-Metwally, Mayada Tarek

**Affiliations:** Computer Science Department, Faculty of Computers and Information, Mansoura University, Mansoura, Dakahlia, Egypt; Northeast Normal University, CHINA

## Abstract

The kidney exchange programs bring new insights in the field of organ transplantation. They make the previously not allowed surgery of incompatible patient-donor pairs easier to be performed on a large scale. Mathematically, the kidney exchange is an optimization problem for the number of possible exchanges among the incompatible pairs in a given pool. Also, the optimization modeling should consider the expected quality-adjusted life of transplant candidates and the shortage of computational and operational hospital resources. In this article, we introduce a bio-inspired stochastic-based Ant Lion Optimization, ALO, algorithm to the kidney exchange space to maximize the number of feasible cycles and chains among the pool pairs. Ant Lion Optimizer-based program achieves comparable kidney exchange results to the deterministic-based approaches like integer programming. Also, ALO outperforms other stochastic-based methods such as Genetic Algorithm in terms of the efficient usage of computational resources and the quantity of resulting exchanges. Ant Lion Optimization algorithm can be adopted easily for on-line exchanges and the integration of weights for hard-to-match patients, which will improve the future decisions of kidney exchange programs. A reference implementation for ALO algorithm for kidney exchanges is written in MATLAB and is GPL licensed. It is available as free open-source software from: https://github.com/SaraEl-Metwally/ALO_algorithm_for_Kidney_Exchanges.

## Introduction

The number of terminal-stage renal patients has significantly risen around the world. In this final stage of disease, kidneys fail to meet patient’s life needs, it may eventually lead to a complete kidney failure. The only available treatment for those patients is a kidney transplant operation [1]. There are two sources for kidneys transplantation. The first way is through deceased donors. However, this way can not satisfy the increasing number of kidney patients, by the end of 2016, there is a waiting list of more than 96,000 patients who need a kidney transplant operation in United States [2]. The other kidney transplantation way is through willing living donors. Recently, the number of kidney transplantation through willing living donors has increased [1]. Transplanting kidneys from living donors have higher rate of success than deceased donors [3].

It is common that a willing donor is ready to donate his kidney, but his blood and tissue types are not compatible with the patient (incompatible donor-patient pair) [4]. Having a set of incompatible pairs, there is a possibility that a donor of a pair i is compatible with the patient of a pair j and vice versa. This case achieves mutual benefits among donor-patient pairs. Therefore, the goal of a kidney exchange program, also called Kidney paired donation(KPD), is to maximize the number of possible matches among the incompatible pairs in a given kidney exchange pool. [3], and hence enhancing the success of kidney transplantation through living donors.

Generally, KPD could be applied in static or dynamic environment [4]. In static environment, KPD attempts to find possible matches that are found in the current pool, i.e., providing local solutions without taking future events in consideration. In dynamic environment, pool size periodically changes, existing pairs can leave because patient passes away or he successfully receives a kidney (cured), or new pairs can arrive. KPD attempts to match as many pairs by taking into account future events. In this case, KPD first searches for a match for hard-to-match pairs (i.e., patients with a rare blood type) and postpones easy-to-match pairs to the next round, which will increase the number of kidney transplantation and save more lives.

Solving a kidney exchange problem efficiently is still an active area of research. A standard tree search algorithm is used as one of the early starting solution to this problem and has a memory space limitation when the size of the patient-donor pairs is increased [5]. Other researchers in this field attempt to solve this problem by formalizing the kidney exchange as an optimization problem. Integer programming approach is used to find the optimal matches among incompatible pairs. The goal of the optimization process is to maximize the number (quantity) or utility (quality) of transplants. In [1, 5–9], Integer programming is used to solve some variants of kidney exchanges with/without chains and make a bound on the pool size or the number of constraints in the matching process. Also, kidney exchange problem can be viewed as a travelling salesperson problem and can be solved using a recursive integer programming formulation [10].

Despite the advantages introduced by the variant techniques that rely on integer programming approach, no stochastic-based algorithm is introduced and used to solve the kidney exchange problem efficiently. This opens a race to solve this problem using different types of heuristics. In [11], authors present a novel model for enhancing matching in the dynamic environment of networked markets. The proposed model has a set of parameters to learn. Inspired by this model, a new trend of kidney exchange algorithms based on stochastic approaches is introduced [4]. This approach employs a learning strategy for optimal solution searching. The methods in [4, 12] used genetic algorithm (GA) with different represented solutions. GA is utilized to search for the best matches in a given KPD pool. The proposed methods achieve reasonable results in terms of the number of resulting transplants compared to the best known methods. However, the computational running time needed to provide these solutions is significantly long, which is impractical to real life situations. Also, due to the large search space, classical GA searching strategy surfers from the local optimal solution, which in turn affects the overall performance. Moreover, the adopted methods are not flexible to be applied in the dynamic environment [4].

In the view of these limitations, this paper presents a novel stochastic based method for kidney exchanges. Ant Lion Optimization (ALO) algorithm is proposed to optimally find the possible matches in a kidney exchange pool. ALO is a recent met-heuristic algorithm that is computationally less expensive than other methods. It provides an improving search strategy in terms of exploration, exploitation, and convergence [13]. A post-processing stage follows the Ant lion optimization algorithm is also introduced to enhance the utility of the resulting exchanges. Moreover, the proposed method can be easily adopted in the dynamic kidney exchanges.

The rest of this paper is organized as follows: a background information about the kidney donation programs, and a brief introduction of the general ant lion optimization algorithm are given in Section 1. The proposed ant lion optimization for kidney exchanges is introduced in Section 2. Experimental results are presented in Section 3. Finally, Section 4 concludes this paper with a brief discussion of our produced results.

## 1 Background

### 1.1 Kidney paired donations

Kidney transplantation is the only hope for curing patients with terminal-stage renal disease. The increasing number of waiting patients on the scheduled list for transplantation and stagnant rate of kidney donations could break this hope of saving more lives. Some patients have relatives that are willing to donate their kidney but they have incompatible blood or tissue types. These patients constitute donor-patient couples and create a pool of pairs that are able to exchange their kidneys based on their compatibility, allowing what is called recently kidney paired donation program [14].

When this program was first executed, the hospitals arranged the exchange locally among their patients. With the increasing number of patients, the kidney shortage, and the availability of databases and registries, the organization of kidney exchange programs are globally transformed and controlled by national and international centers. The National Kidney Registry (NKR) [15], the Alliance for Paired Donation (APD) [16, 17], and the United Network for Organ Sharing (UNOS) [18] are among the emerging organizations for kidney exchange programs on a large scale in the USA. Other emerging centers are reported from several counties such as UK [19], Canada [20], and Australia [21].

One of the major goals of KPD programs is allowing more kidney exchange to take place while improving the efficiency of kidney matching by searching for a better match in less waiting and dialysis time (i.e., the kidney donation from a living person lasts longer than the kidney from a deceased one and the long waiting list causes that some patients are died and others are becoming too ill for surgery).

The two important factors that play a key role on a kidney matching decisions are ABO blood types and Human Leukocyte Antigen tissue type, HLA. There are four blood types a patient could have, A, B, AB, or O. The matching compatibility rules between the patients and donors according to their blood types are described in Table 1. Donors with blood type O, a general donor, can donate to any patients while patients with type AB, general recipient, can receive a kidney from any donors. HLA tissue type consists of six proteins that are required to match between the donors and patients to successfully transplant a kidney. Moreover, there is a possibility of the positive cross match between the patient and the donor but the patient’s body has antibodies that target the donor’s HLA, which precludes the kidney transplantation [22].

**Table 1:** Compatibility rules based on ABO blood type.

**Donor/Patient**	A	B	AB	O
A	✓		✓	
B		✓	✓	
AB			✓	
O	✓	✓	✓	✓

**Fig 1:**
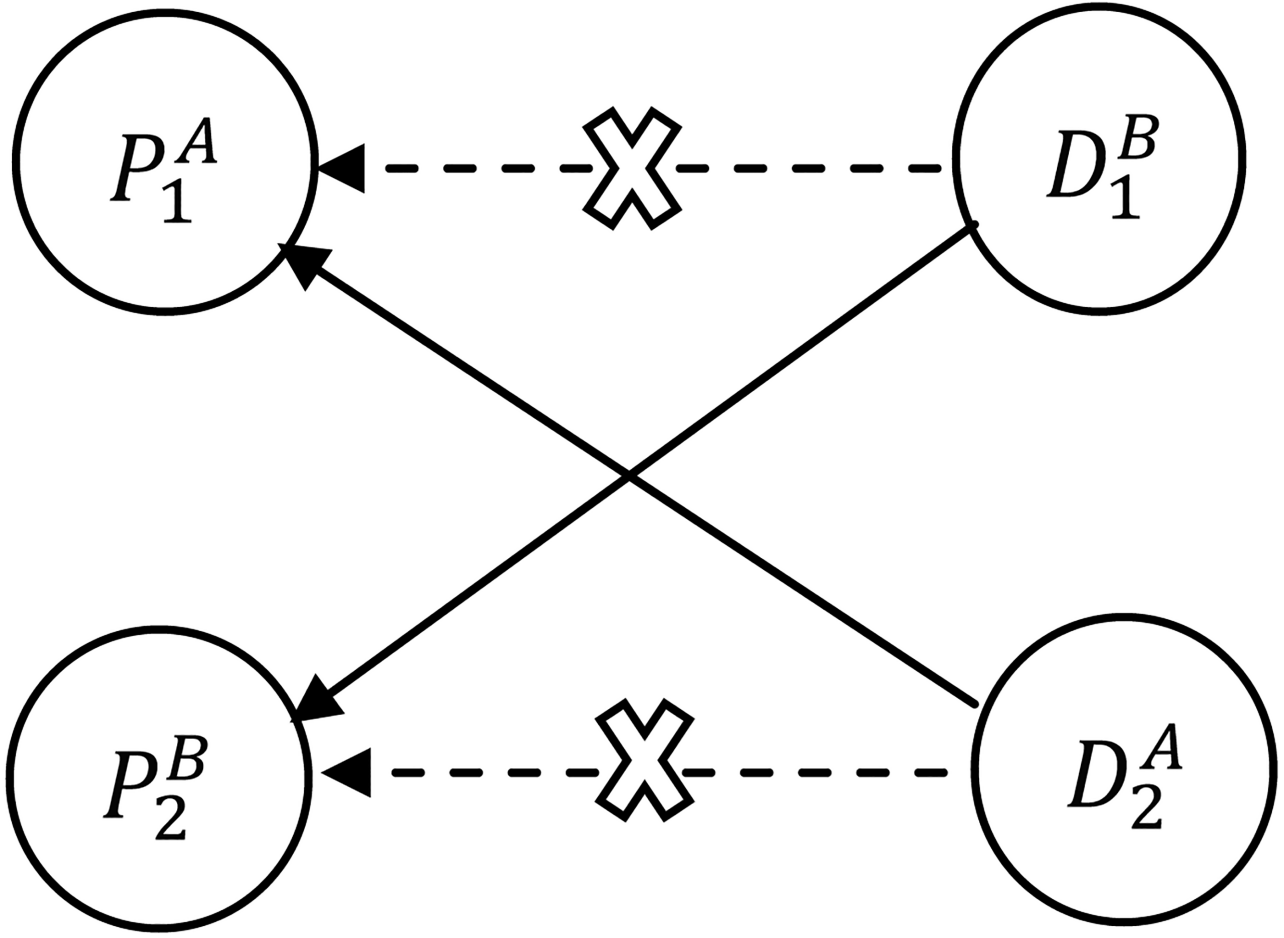
Two-cycle exchange. ${\text{P}}_{1}^{\text{A}}$ blood type is incompatible with his donor ${\text{D}}_{1}^{\text{B}}$, the incompatibility is represented by dotted lines. The two incompatible pairs can swap their kidneys as illustrated by solid lines.

The number of donor-patient pairs in a given pool shapes the complexity of kidney exchange problem. The simple 2-way or 2-cycle exchange is represented by alternating directed cycle of two incompatible donor-patient pairs where the donor of the first incompatible pair gives a kidney to the patient of the second incompatible pair and vise versa. (Fig 1 illustrates 2-way or 2-cycle exchange). The size of exchange cycle can be scaled up to three or more by including more donor-patient pairs in the exchange pool. Also, the pool can be considered as a dynamic i.e., on-line, if the donor-patient pairs are appeared and expired over a time, otherwise it is static or off-line [23, 24].

The long exchange cycle, k-cycle, where k ≥ 3 allows more options for kidney exchange and consequently better optimization for solving the kidney matching problem. When the number of pairs in an exchange pool is not fixed to a number n, i.e., k = n, the problem is considered as an assignment problem with a polynomial solving time [5]. In the reality, the number of pairs in the pool and consequently the length of an exchange cycle should be bounded for the two reasons. First, all operations involved in a cycle must be performed simultaneously to guarantee the commitment of kidney exchange process between the incompatible pairs. This will introduce additional overhead on the hospitals with limited resources. Second, the long exchange cycle might have unpredictable events that cause the cancellation of the exchange process. For example, the patient death or the new incompatibility issues that are appeared between the pairs on the last minute can preclude the exchange. The kidney matching problem with two-cycle can be solved optimally in a polynomial time using Edmonds maximum cardinality matching algorithm while the large scale k-cycle is difficult to solve and considered as NP-complete problem [25].

**Fig 2:**
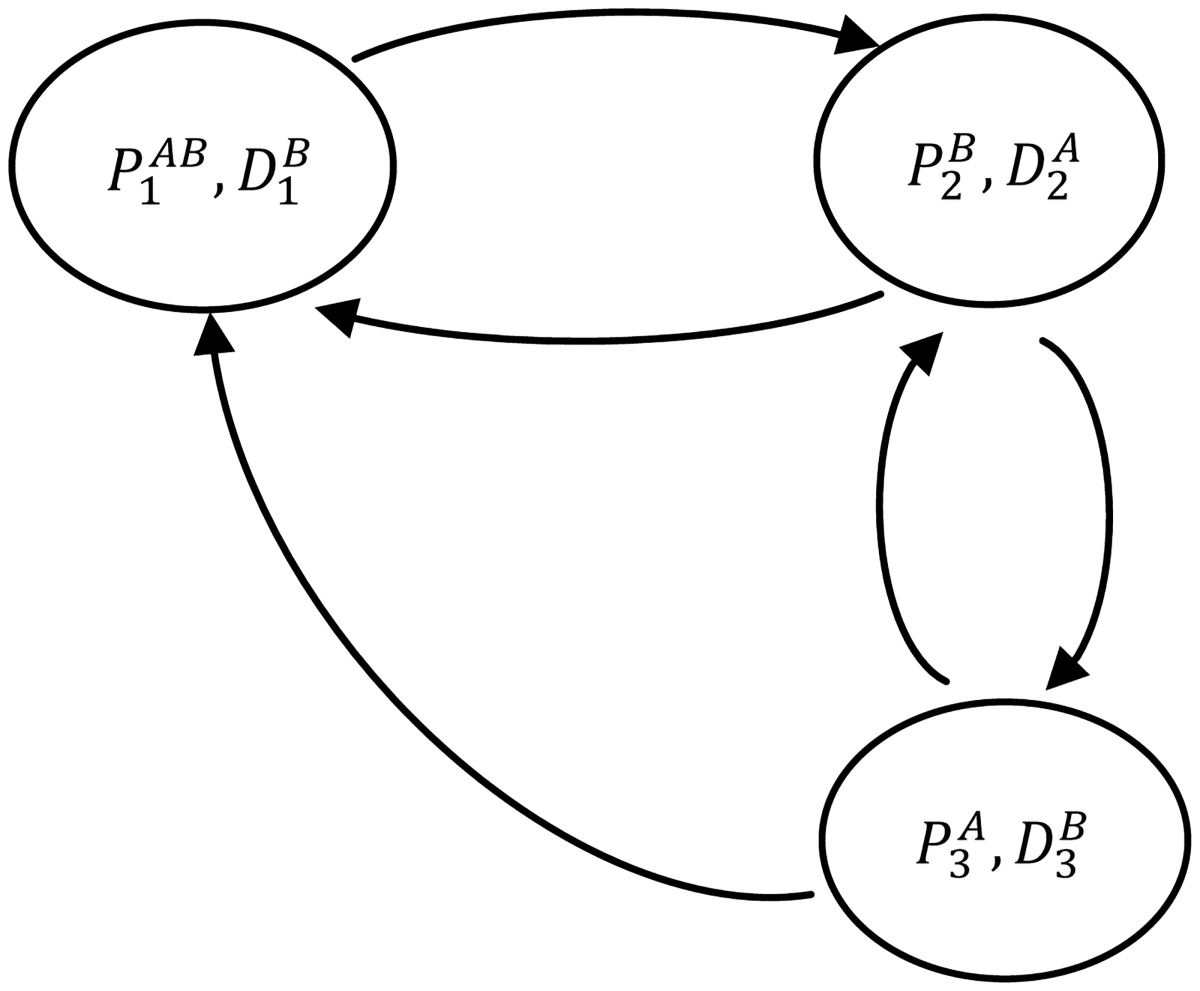
A directed graph representation of three-cycle exchange. The solid arcs represent the potential swaps, i.e. the two pairs (${\text{P}}_{1}^{\text{AB}}$,${\text{D}}_{1}^{\text{B}}$) and (${\text{P}}_{2}^{\text{B}}$,${\text{D}}_{2}^{\text{A}}$) can exchange their kidneys.

The kidney exchange problem is formulated as a directed graph with the nodes being the exchange pairs in the pool. The directed edges encode the constrains of the compatibility matches among the pairs (Fig 2 shows a directed graph of three-cycle exchange). The goal of any kidney exchange program is to find a tour in the graph that includes every edge exactly once and provides mutual benefits. This type of tour is called a feasible tour or a feasible cycle.

**Fig 3:**
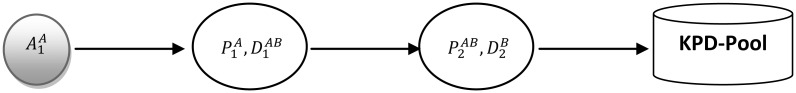
A NEAD exchange graph. A chain starts with an altruistic donor and ends with a bridge donor assigned to the first compatible patient on the next round.

There are some variants of kidney exchange problem formulation such as initiating the exchange by a donor who is not associated with any patient and is willing to donate a kidney, i.e., altruistic donor. The exchange starts with an altruistic donor is called a chain or non-directed exchange. The altruistic donor donates a kidney to a patient of one pairs in the kidney exchange pool and the associated patient’s donor will exchange a kidney with the first compatible patient on the deceased donors waiting list. Never-Ending Altruistic Donor, NEAD, is a variant of altruistic kidney exchange where the recipient’s donor is not associated with any waiting list but donates a kidney to a compatible patient. The patient’s incompatible donor continues this cascading chain by donating a kidney in the same way and so on (theoretically, this cascading chain never ends) [26] (Fig 3 illustrates an example of NEAD). Another variant of a kidney exchange problem is including of compatible pairs and multiple donors, which will increase the possibility of finding more cycles and chains with many incompatible pairs in the kidney exchange pool [5, 27].

### 1.2 Ant Lion Optimization algorithm

Inspired by the rich nature of biological phenomena, scientists mathematically introduce novel algorithms for modeling the real world optimization problems [28]. These bio-inspired algorithms adopt stochastic procedures to overcome the limitations of traditional techniques for solving the optimization problems [29, 30]. Recently, a new bio-inspired algorithm, namely, Ant Lion Optimization (ALO) is introduced by modeling the nature chasing behavior of ant lions to catch its prey, usually ants [13].

**Fig 4:**
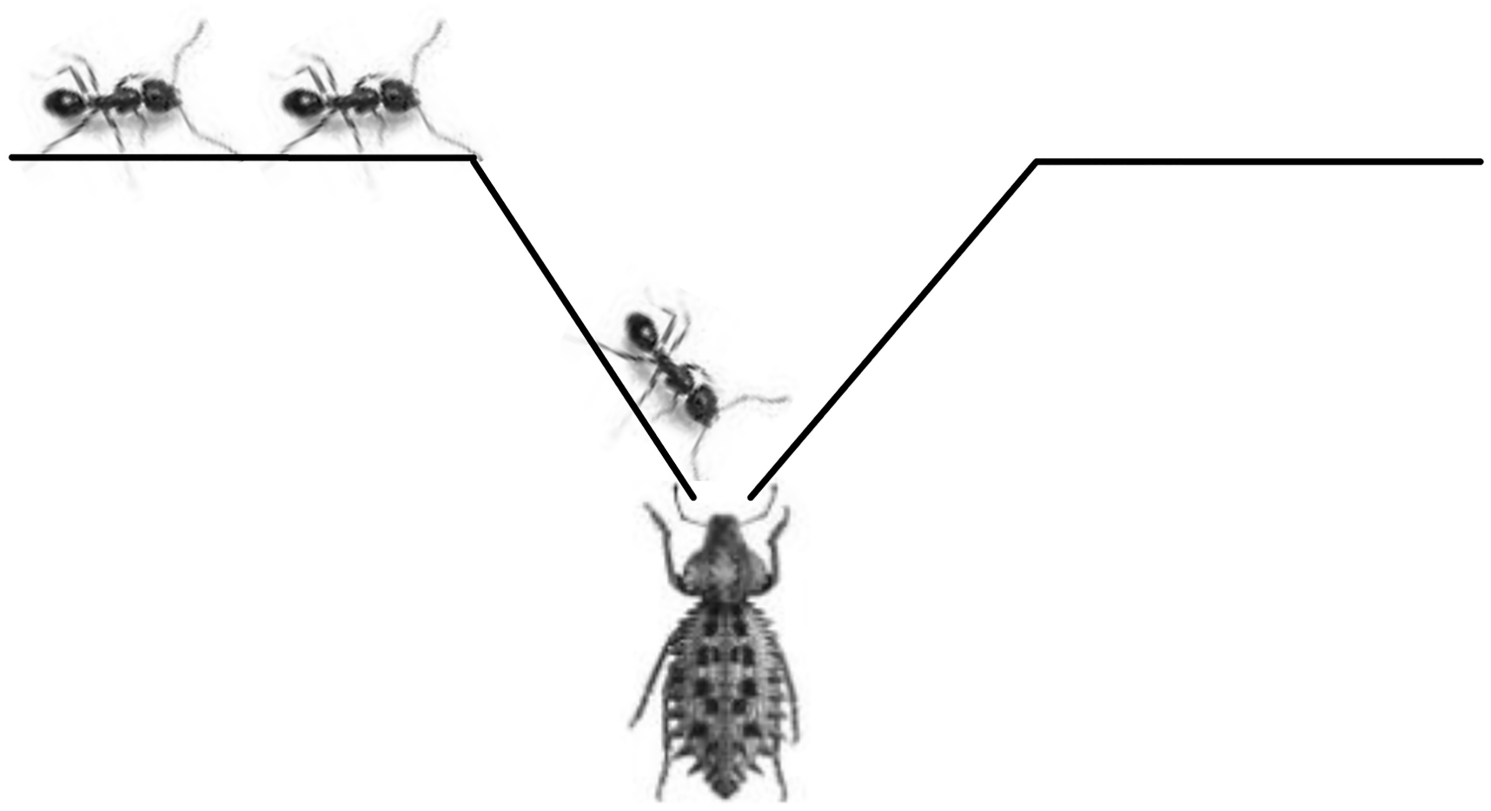
A hunting behavior of Ant lions.

Ant lions move in circular paths to drill cone-shaped holes in the sand to catch their prey. They hide at the bottom of the holes waiting for hunting insects as illustrated in Fig 4. The bigger holes have higher probabilities for catching a prey and are usually created by a fitter elite ant lion. Once an insect is caught, Ant lions pull it under the soil by throwing the sand towards the outer edge of the hole using its big jaw, which prevents the insect from escaping. Then, ant lions consume the prey, throw the leftovers outside the hole, rebuild the hole, and get ready for the next hunt. The fitness of ant lions and the quality of created holes are improving during each hunt [13].

The ants in ALO algorithm represent the possible random solutions for a given problem in the search space and the ant lions drill holes in the ground to catch and consume ants. The hunting ability of ant lion is encoded in the objective function and is optimized according to the interaction between ants and ant lions. There are some rules to consider when modeling the optimization problem using the nature of hunting behavior of ant lions. The ants in ALO generate random walks in the search space, which are affected by the dimensions of all ants and the ant lions created holes. The cone-shaped holes sizes are proportional to the fitness of each ant lion, i.e. the fitter ant lions can build bigger holes and hence have a higher possibility to catch a prey. The ants can be consumed by any ant lion or the elite one and the range of their random walks is decreased adaptively when the ant lion slides the ants towards the bottom of the hole. Consequently, the consuming ant lions become fitter than ants, take their positions, and rebuild the hole for improving their chance for catching other ants [13, 31–33].

In terms of clinical application of ALO in the kidney exchange space, the ants represent the possible kidney exchanges in a given pool. Maximizing the hunting ability of ant lion mimics increasing the number of matches among the pairs according to the defined optimization function. ALO optimally explores the search space and lead to better optimized solutions and convergence rates [33].

The ants and hidden ant lions are located in the same search space and their positions during the optimization process are stored in M_ant_ and M_antlion_ matrices.

$$\begin{array}{c} M_{ant}=[\begin{array}{cccc} A_{1,1} & A_{1,2} & \cdots & A_{1,d}\\ A_{2,1} & A_{2,2} & \cdots & A_{2,d}\\ \vdots & \vdots & \ddots & \vdots \\ A_{n,1} & A_{n,2} & \cdots & A_{n,d} \end{array}] \end{array}$$ (1)

$$\begin{array}{c} M_{antlion}=[\begin{array}{cccc} AL_{1,1} & AL_{1,2} & \cdots & AL_{1,d}\\ AL_{2,1} & AL_{2,2} & \cdots & AL_{2,d}\\ \vdots & \vdots & \ddots & \vdots \\ AL_{n,1} & AL_{n,2} & \cdots & AL_{n,d} \end{array}] \end{array}$$ (2)

The n and d denote the ant’s /ant lion’s number and their dimension respectively. A_i,j_ and AL_i,j_ represent the value of j^th^ dimension of i^th^ ant/ant lion respectively. The ant’s dimension encodes the number of variables in the optimization problem that affect the current problem solution, i.e. current ant. Each row in M_ant_ represents a solution to a given optimization problem according to d decision variables. The ants/ant lions are evaluated using objective function f and their evaluation values are stored in M_OA_ and M_OAL_ matrices [13, 31–33].

$$\begin{array}{c} M_{OA}=[\begin{array}{c} f(A_{1,1},A_{1,2},\cdots ,A_{1,d})\\ f(A_{2,1},A_{2,2},\cdots ,A_{2,d})\\ \cdots ,\cdots ,\cdots ,\cdots \\ f(A_{n,1},A_{n,2},\cdots ,A_{n,d}) \end{array}] \end{array}$$ (3)

$$\begin{array}{c} M_{OAL}=[\begin{array}{c} f(AL_{1,1},AL_{1,2},\cdots ,AL_{1,d})\\ f(AL_{2,1},AL_{2,2},\cdots ,AL_{2,d})\\ \cdots ,\cdots ,\cdots ,\cdots \\ f(AL_{n,1},AL_{n,2},\cdots ,AL_{n,d}) \end{array}] \end{array}$$ (4)

Modeling any optimization problem using ALO algorithm requires defining the following operations:

- Building ant lion traps: each ant is assumed to be caught by one ant lion. The selection of an ant lion is based on its hunting ability that is encoded in the objective function. The fitter ant lions can build bigger holes and have higher ability to catch a prey. A Roulette wheel method gives high chances for the fitter ant lions to be selected for catching ants.
- Generating ants’ random walks: naturally, ants move in random paths searching for their food. Therefore, their movements in the i^th^ dimension of a d-dimensional space, ${\text{X}}_{\text{i}}^{\text{n}}$, is modelled using random cumulative sum function defined by Eq (5):
  $$\begin{array}{c} {\text{X}}_{\text{i}}^{\text{n}}=\left[0,\text{cumsum}\left(2\text{r}\left({\text{n}}_{1}\right)-1\right);\text{cumsum}\left(2\text{r}\left({\text{n}}_{2}\right)-1\right);\ldots \text{cumsum}\left(2\text{r}\left({\text{n}}_{\text{N}}\right)-1\right)\right] \end{array}$$ (5)
  where: n denotes the current iteration, N represents the total number of iterations, cumsum returns the cumulative sum, and r(n) is a random function returns value ‘1’ if a randomly generated number is greater than ‘0.5’ and returns ‘0’ otherwise.
${\text{X}}_{\text{i}}^{\text{n}}$ encodes the changes (movements) that happen to the current solution (ant) by including the i^th^ decision variable (dimension) in the optimization problem.
  In order to bound ant’s movements inside the search space, values are normalized using a min-max normalization function defined by Eq (6):
  $${\text{X}}_{\text{i}}^{\text{n}}=\frac{\left({\text{X}}_{\text{i}}^{\text{n}}-{\text{a}}_{\text{i}}\right)\times \left({\text{d}}_{\text{i}}-{\text{c}}_{\text{i}}^{\text{n}}\right)}{\left({\text{b}}_{\text{i}}^{\text{n}}-{\text{a}}_{\text{i}}\right)}+{\text{c}}_{\text{i}}$$ (6)
  where: a_i_ and b_i_ denote the minimum and maximum random walk of the i^th^ variable, respectively, and ${\text{c}}_{\text{i}}^{\text{n}}$ and ${\text{d}}_{\text{i}}^{\text{n}}$ represent the minimum and maximum of the i^th^ variable at n^th^ iteration, respectively.
- Chasing ants in the holes: once the ant is trapped in the ant lion’s hole, its movement is restricted by the position of the hunting ant lion (antlion_j_). This is modelled by Eqs (7), and (8):
  $${\text{c}}_{\text{i}}^{\text{n}}={\text{c}}^{\text{n}}+\text{ant}{\text{lions}}_{\text{j}}^{\text{n}}$$ (7)
  $${\text{d}}_{\text{i}}^{\text{n}}={\text{d}}^{\text{n}}+\text{ant}{\text{lions}}_{\text{j}}^{\text{n}}$$ (8)
  where: c^n^ and d^n^ represent the minimum and maximum of all variables at n^th^ iteration, respectively. ${\text{c}}_{\text{i}}^{\text{n}}$ and ${\text{d}}_{\text{i}}^{\text{n}}$ denote the minimum and maximum of all variables for ant_i_, respectively.
- Sliding trapped ants: ant lions slide ants towards their created traps to prevent them from escaping. This process is mathematically modeled by iteratively decreasing the lower and upper bounds of ant’s random walk, Eqs (9) and (10):
  $${\text{c}}^{\text{n}}=\frac{{\text{c}}^{\text{n}}}{\text{I}}$$ (9)
  $${\text{d}}^{\text{n}}=\frac{{\text{d}}^{\text{n}}}{\text{I}}$$ (10)
  where: c^n^ and d^n^ represent the minimum and the maximum of all variables at n^th^ iteration respectively, and i is a ratio defined by Eq (11):
  $$\text{I}=10^{\text{w}}\frac{\text{n}}{\text{N}}$$ (11)
  where: w denotes a dynamic parameter used to control the search exploitation level.
- Catching ants and hole re-building: The final step of hunting process is to eat the trapped ants and re-building the previously used hole. Mathematically, this process is modelled by comparing the fitness of consumed ants with their hunting ant lions. If an ant becomes fitter than its corresponding ant lions, it means that it is inside the hole and it is about to be consumed. Accordingly, Ant lions replace their positions with the consumed ants looking for increasing their chances for the next hunt. This is defined by Eq (12):
  $${\text{Antlions}}_{\text{j}}^{\text{n}}={\text{Ant}}_{\text{i}}^{\text{n}}\;\text{If}\;\text{f}\left({\text{Ant}}_{\text{i}}^{\text{n}}\right)>\text{f}\left({\text{Antlions}}_{\text{j}}^{\text{n}}\right)$$ (12)
  where: ${\text{Antlions}}_{\text{j}}^{\text{n}}$ represents ant lion’s position j at n^th^ iteration, and ${\text{Ant}}_{\text{i}}^{\text{n}}$ represents ant’s position i at n^th^ iteration, assuming maximization of the objective function.
- Maintaining the best solution: ALO adapts elitism strategy in order to preserve the best obtained solution across different iterations. The position of the fittest ant lion, i.e. elite ant lion, is used to guide the random walk of each ant as represented by Eq (13):
  $${\text{Ant}}_{\text{i}}^{\text{n}}=\frac{\left({\text{R}}_{\text{A}}^{\text{n}}+{\text{R}}_{\text{E}}^{\text{n}}\right)}{2}$$ (13)
  where: ${\text{R}}_{\text{A}}^{\text{n}}$ denotes random walk around the selected ant lion, and ${\text{R}}_{\text{E}}^{\text{n}}$ denotes the random walk around the elite ant lion at n^th^ iteration.

## 2 The proposed ALO algorithm for kidney exchanges

**Fig 5:**
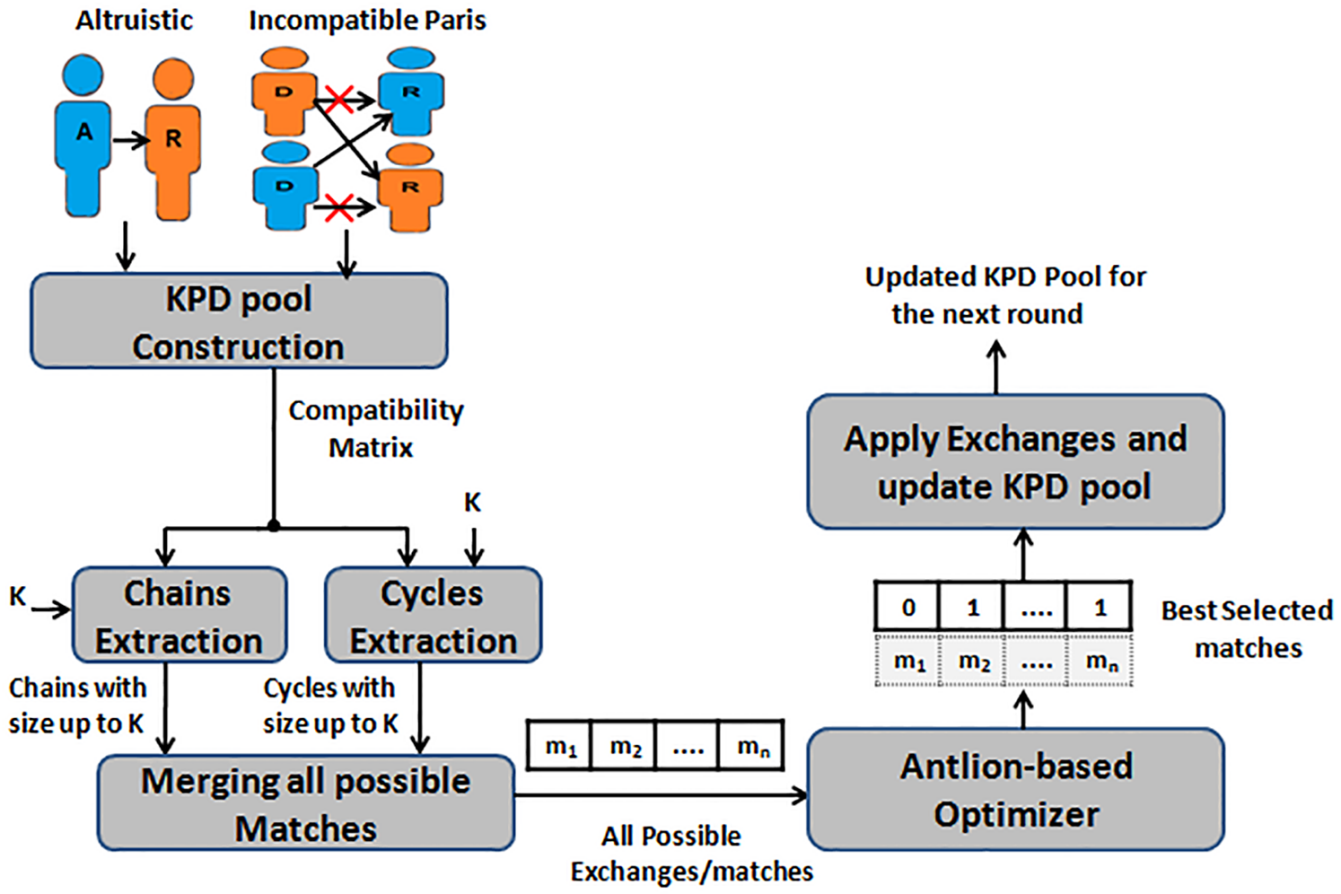
KPD using Binary Ant Lion Optimization algorithm.

In our proposed method, kidney exchange is formulated as a combinatorial optimization problem. The goal is to maximize the exchange utility while satisfying the problem constraints. Feasible exchanges are only allowed (i.e. nodes involved in a given exchange can not be used in any other exchanges). Also, the length of exchange is restricted to the maximum allowed size for chains and cycles, which satisfies the hospital resources. The proposed methodology utilizes the ant lion optimization algorithm to select the optimal exchanges in a manner similar to wrapper methods in feature selection algorithms. Fig 5 gives a schematic description of the proposed ALO algorithm for kidney exchanges.

**Fig 6:**
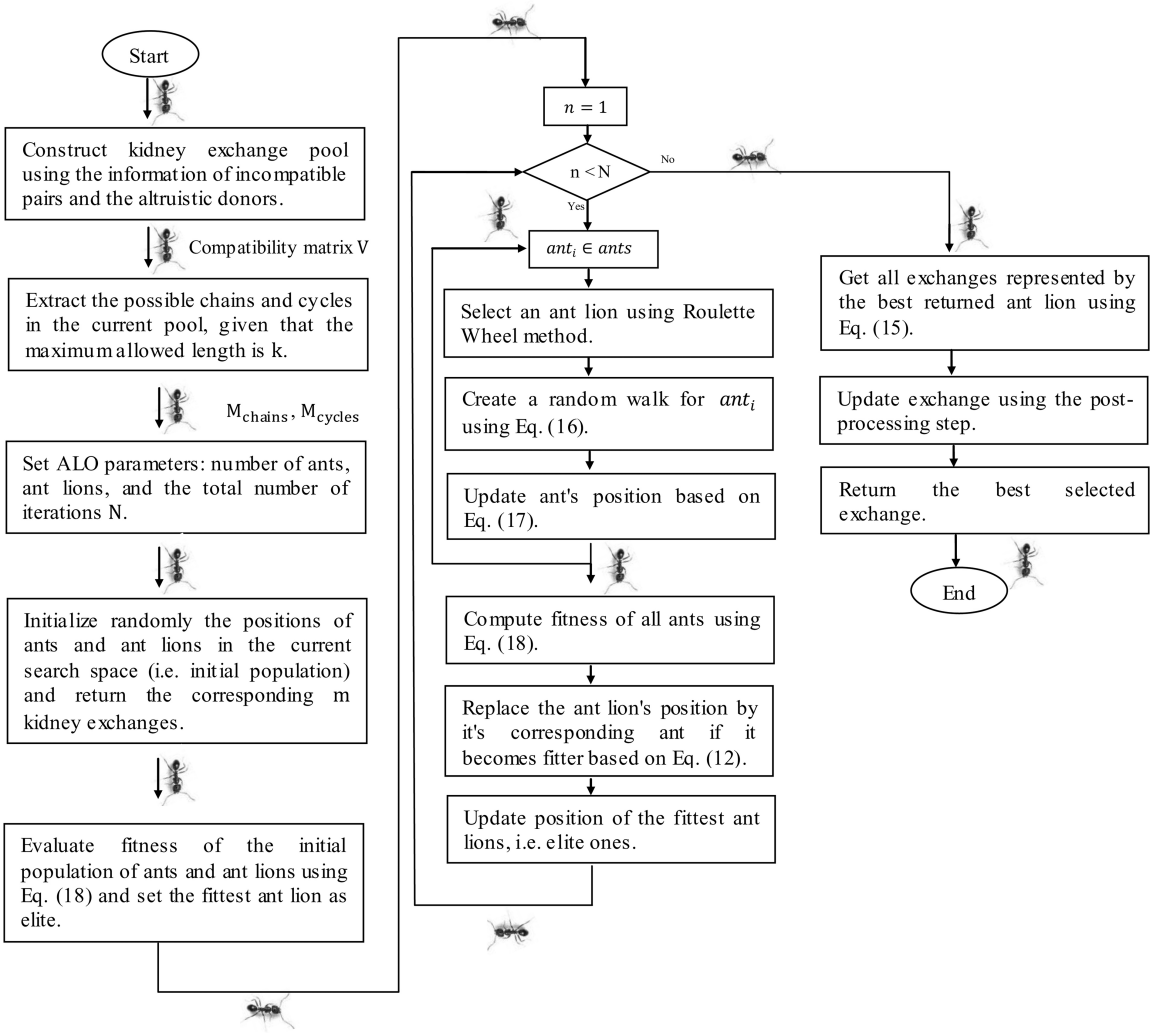
A flow chart of the proposed ALO algorithm for kidney exchanges.

As depicted in Fig 5, the proposed method is composed of three main stages: First, constructing the KPD pool using the candidate pairs, the altruistic donors, and the compatible relations between them. Second, extracting all possible chains and cycles in the current KPD pool given that the maximum allowed length is k. Finally, providing all of the possible matches to the ant lion optimizer to select the best matches/ exchanges, which maximizes the number of patients receiving a kidney. After each successfully kidney transplants, KPD pool is updated and ready for a new round of kidney exchange. Fig 6 summarizes the steps used in our algorithm while the following sections explain each step in detail.

### 2.1 KPD pool construction

Giving a list of incompatible pairs nodes n, and altruistic donors nodes A, the compatibility matrix V, is generated, as explained by Algorithm (1). This stage is implemented in every new round or upon the arrival/ departure of new nodes to KPD pool.

**Algorithm 1** KPD Pool Construction.

**Input**:

  • The Altruistic donors set A: {a_1_,..,a_u_}.

  • A set of incompatible pairs N: {n_1_,..,n_z_} where n_i_: denotes the donor/ patient pair $\left({\text{D}}_{1}^{\text{A}},{{\text{P}}_{1}}^{\text{B}}\right)$.

  • The compatible relations set *C*: {c_1_,..,c_l_} where c_i_: (n_i_, n_j_, w_i,j_) indicates that donor of node n_i_ is compatible with patient of node n_j_ with utility value w_i,j_.

**Output**: Compatibility matrix, a square matrix indicates the computability relations in the current pool.

1: Initialize v(i, j) = 0, ∀{i, j}, where: i, j ≤ u + z.

2: **for each** c_i_ ∈ C **do**

3:  x ← n_i_.

4:  y ← n_j_.

5:  v(x, y) = w_i,j_.

6: **end for**

7: Return compatibility matrix V.

### 2.2 Chains and cycles extraction

Chains and cycles can have different lengths. However, it is not recommended to have a large size of cycles and/or chains. Practically, exchanges are executed simultaneously to guarantee the commitment of kidney exchange process between the incompatible pairs. Therefore, chains’ and cycles’ length are restricted to the available hospital resources. In our proposed methodology, there is a dynamically selected parameter k denotes the maximum allowable length for chains and cycles. Algorithms (2) and (3) present the process of chains and cycles extraction respectively. The chains and cycles can be extracted sequentially or in parallel.

**Algorithm 2** Chains Extraction.

**Input**:

  • Compatibility matrix V, if all w_i,j_ = 1, v_i,j_ ∈ {0, 1}.

  • Maximum allowed length for chains, k.

**Output**: All possible chains up to the maximum length.

1: M_chains_ ← Φ.

2: Find the altruistic nodes in current pool, A.

3: **for each** n_i_ ∈ A **do**

4:  t = 1.

5:  **while** t < k **do**

6:   Generate all possible compatible sequence of nodes starting from root node n_i_ to nodes at depth = t.

7:   Save all resulting paths in X, A path is the sequence of nodes: {n_i_, …, n_t_}.

8:   For each x_i_ ∈ X,

9:   M_chains_ ← {M_chains_ ∪ x_i_}.

10:   t = t + 1.

11:  **end while**

12: **end for**

13: Return all possible chains M_chains_.

As stated in [4], one of the limitations for stochastic-based optimization methods for kidney exchanges is the large computational running time needed for producing the optimal feasible exchanges. Extra processing is needed during evolution for restricting exchanges to the only feasible solutions. In order to decrease the overall running time, we suggested the restriction of search space to the initial extracted chains and cycles from KPD pool. The chains/cycles extraction is performed only once in our algorithm as a pre-processing stage, which reduces the overall algorithm running time.

**Algorithm 3** Cycles Extraction.

**Input**:

  • Compatibility matrix V, where if all w_i,j_ = 1, v_i,j_ ∈ {0, 1}.

  • Maximum allowed length for cycles, k.

**Output**: All possible cycles up to the maximum length.

1: M_cycles_ ← Φ.

2: t = 2.

3: **while** t ≤ k **do**

4:  **for each** n_i_ ∈ N: incompatible pair nodes in the current pool **do**

5:   Generate all possible compatible sequence of nodes starting from root node n_i_ to nodes at depth = t.

6:   Save all resulting paths in X, A path is the sequence of nodes: {n_i_, …, n_t_}.

7:   For each x_i_ ∈ X,

8:   **if** n_i_ = n_t_ **then**

9:    **if** x_i_ ∉ M_cycles_ **then**

10:     M_cycles_ ← {M_cycles_ ∪ x_i_}

11:    **end if**

12:   **end if**

13:  **end for**

14:  t = t + 1.

15: **end while**

16: Return all possible cycles M_cycles_.

### 2.3 Binary Ant Lion Optimization algorithm

In our proposed method, each individual is represented by a vector with a dimension equals to the total number of exchanges extracted from the given KPD pool. Usually individuals of a classical Ant Lion Optimization algorithm are represented by a continuous valued vectors. Since our representation of KPD problem is formulated as a combinatorial optimization problem, vector values are restricted to the binary range [0, 1].

Many binarization methods have been proposed to adapt continuous meta-heuristic optimization algorithms for solving binary problems [34]. Great Value Priority (GVP), is a binarization method used to map the individual’s representation from a continuous space to the binary one. GVP generates a permutation sequence P based on sorting the original individual’s vector and assigning to P the maximum values in order. The process is repeated for all dimensions of all individuals and P is binarized using Eq (14):

$${\text{Y}}_{\text{i}}=\{\begin{array}{cc} 1 & {\text{P}}_{\text{i}}\geq P_{i+1}\\ 0 & \text{Otherwise} \end{array}$$ (14)

The mapping process of GVP reflects a priority order relation, which is suitable for our formulation of KPD problem. Inspired by this priority binary mapping, we initialized the positions of ants and ant lions using the previous mapping procedures. The ALO with a binary vector representation is called Binary Ant Lion Optimization algorithm. The solution represents the best selected matches/exchanges in the complete set of M exchanges computed by Eq (15):

$${\text{m}}_{\text{i}}=\{\begin{array}{cc} \text{M}(\text{i}) & {\text{Y}}_{\text{i}}=1\\ \Phi & {\text{Y}}_{\text{i}}=0 \end{array}$$ (15)

where M represents the complete set of pool’s exchanges produced by the pre-processing stage.

In order to maintain the binary representation of ALO’s individual, we replaced the random walk function (Eq (5)) by a mutation operation applied to the given ant lion (Eq (16))

Antlion(i)=¬Antlion(i) (16)

where Antlion represents the positions vector for the input ant lion, and i is a randomly selected dimension of the ant lion. Each ant updates its position based on a crossover operation computed by Eq (17):

$${\text{Ant}}^{\text{d}}=\{\begin{array}{cc} {\text{RE}}^{\text{d}} & r^{d}<0.5\\ {\text{RA}}^{\text{d}} & \text{Otherwise} \end{array}$$ (17)

where RE and RA represent the mutated positions for the elite ant lions and a randomly selected ant lion, respectively. Ant^d^ represents the new ant position at dimension *d*, r is a vector contains randomly generated numbers drawn from a uniform distribution in the range [0, 1].

The goal of the utilized Binary Ant Lion Optimization algorithm is to maximize the kidney exchange’s utility. Fitness function F is proposed to map each individual in the search space into a real-valued f, which is used to evaluate the strength of an individual in a given problem. The proposed fitness function is designed according to the following criteria:

- The Altruistic node donates a kidney without taking any benefits in return.
- Every incompatible pairs node in the resulting solution must be involved in the kidney exchange only once:
  - If the node’s donor is used more than once in the resulting exchanges, a penalty value is added.
  - If the node’s receiver is used more than once in the resulting exchanges, a penalty value is added.

The proposed fitness function is defined by Eq (18):

$$\text{maximize}(\sum^{\text{L}}\;\sum^{\text{U}}{\text{w}}_{\text{i},\text{j}})-E$$ (18)

$$\text{E}=({\text{E}}_{1}+{\text{E}}_{2})\lambda$$ (19)

where:

- L denotes the solution length (number of exchanges i.e., chains or cycles represented by the solution);
- U denotes the exchange length (number of compatible relations represented by the cycle or chain);
- i denotes the node’s donor index;
- j denotes the node’s patient index;
- w_i,j_ denotes a utility value for the compatible relation between donor i and patient j (w_i,j_ = 1, if all are equal).
- E_1_ denotes the number of donors used more than once for donating a kidney;
- E_2_ denotes the number of patients used more than once for receiving a kidney;
- λ a penalty value added to maximize error value for solutions, which contain nodes used more than once, λ ≫ pool size.

**Fig 7:**
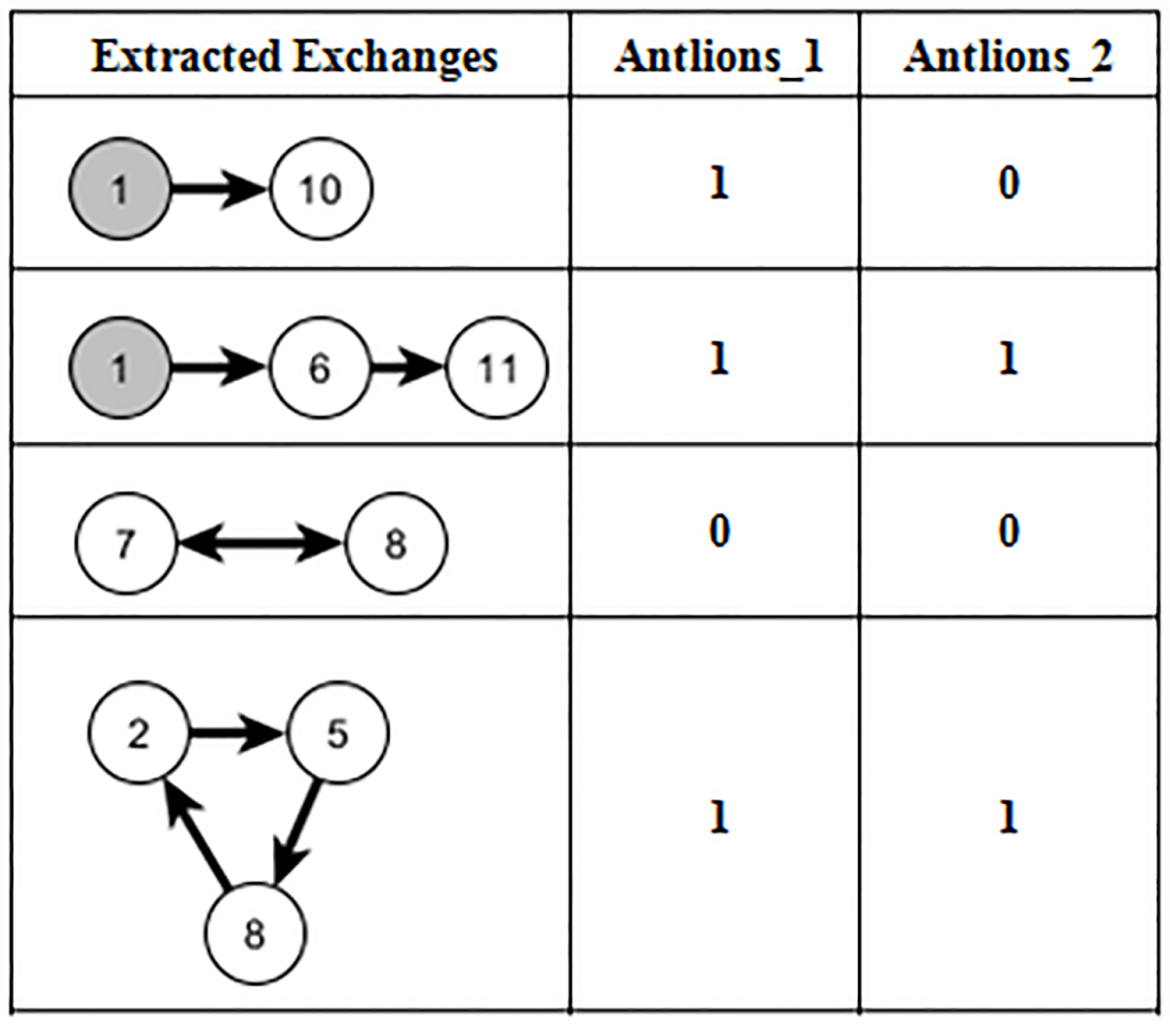
An example of a problem contains four exchanges.

To illustrate the impact of the proposed fitness function, Fig 7 represents an example of exchanges extracted from a KPD pool. The exchanges contain two chains, one cycle of length 2, and one cycle of length 3. The number of ALO dimensions equals four (two chains and two cycles). Fitness with a negative value represents an infeasible exchange. To prevent ALO from producing the infeasible exchanges, a penalty value λ is added to maximize the error value E. As depicted in Fig 7, given antlion_1_ represented by the vector [1101], node (1), is used twice for donating a kidney, therefore, the fitness value of antlions_1_, f = −44, when λ = 50. If the ant lion represents a feasible exchange, as given by antlion_2_ which is represented by the vector [0101], the error value E equals zero and the fitness value indicates the number of transplants, f = 5, which denotes the global optimum solution.

Algorithm (4) presents steps of the proposed Binary Ant Lion optimization algorithm for kidney exchanges. Upon completion of Ant Lion evolution, the resulting exchanges are recommended for kidney transplantations and the KPD pool is updated for preparing the next round of exchanges.

**Algorithm 4** Ant Lion Optimization algorithm for kidney Exchanges.

**Input**: Total number of: ants, antlions, and number of iterations N.

**Output**: Best selected matches/exchanges.

1: For each round, construct KPD pool using Algorithm (1).

2: Extract all possible chains, M_chains_ using Algorithm (2).

3: Extract all possible cycles, M_cycles_ using Algorithm (3).

4: M ← {M_chains_ ∪ M_cycles_}.

5: For each ant_i_ ∈ Ants, Length(ant_i_) ← |M|.

6: For each antlion_i_ ∈ Antlions, Length(antlion_i_) ←|M|.

7: Initialize populations of ants’ and antlions’ positions within the given search domain.

8: Return the exchanges m, represented by ants’ and antlions’ positions where: {m ⊆ M}.

9: Evaluate exchanges using the fitness function defined by Eq (18) and set the corresponding ants’ and antlions’ fitness values.

10: Find the best antlion, i.e. elite.

11: **for** n = 1, 2, …, N **do**

12:  For each ant_i_ ∈ Ants

13:  Select an antlion using Roulette Wheel selection method.

14:  Create a random walk for ant_i_ based on Eq (16) and update it’s position by Eq (17).

15:  Calculate the fitness of all ants.

16:  Replace an antlion with it’s corresponding ant based on Eq (12).

17:  Update the elite if an antlion becomes fitter than the current elite.

18: **end for**

19: Get Exch, all exchanges m_i_ ∈ M, represented by the best returned ant lion based on Eq (15).

20: Update Exch, using the post-processing process defined in Algorithm (5).

21: Return Exch, the best selected exchanges.

Some runs of Ant Lion Optimization algorithm fail to produce the optimal solution and are trapped in a local optimal region. Therefore, a post-processing stage is introduced to maximize the overall exchanges’ utility produced by ALO. Algorithm (5) presents the post-processing step applied to the resulting exchanges.

**Algorithm 5** Candidate Exchanges Post-processing.

**Input**: All possible exchanges M, and candidate exchanges Exch.

**Output**: Updated exchanges, Exch.

1: For each m_i_ ∈ M,

2: **if** m_i_ ∉ Exch **then**

3:  **if** Nodes(Exch) ∩ Nodes(m_i_) = Φ **then**

4:   Exch ← {Exch ∪ m_i_}

5:  **end if**

6: **end if**

7: For each m_i_ ∈ M

8: For each m_j_ ∈ Exch,

9: **if** Utility(m_i_) > Utility(m_j_) **then**

10:  TmpExch ← {Exch − m_j_}

11:  **if** Nodes(TmpExch) ∩ Nodes(m_i_)) = Φ **then**

12:   Exch ← {TmpExch ∪ m_i_}

13:  **end if**

14: **end if**

15: Return updated exchanges, Exch.

In Algorithm (5), Nodes(x) denote nodes represented by exchanges x, and Utility(x) denotes the utility value represented by exchanges x, (if all w_i,j_ = 1, the utility value expresses the number of resulting transplants). The post-processing step searches for extra exchanges, which are not included in the current exchanges. Also, it can maximize the exchanges’ utility or replace the existing exchanges with better ones without violating the solution’s criteria. Giving a KPD pool with M exchanges, a brute force search requires the maximum number of trials equals 2^|M|^, in order to optimally select the best combination of exchanges m ∈ M, while satisfying the problem constrains. The post-processing step that follows the Ant Lion Optimization algorithm requires only the maximum number of trials equals |M| + |M| * |Exch|, where Exch, represents the candidates’ exchanges produced by ALO. The computational running time for all our experiments is explained in detail in the following section.

## 3 Experimental results and discussion

### 3.1 Data sets and experimental settings

We evaluated the performance of the proposed method using six simulated datasets generated from an updated version of Saidman generator [35]. The simulated kidney exchange data has a distribution that approximately mimics the UNOS pool as of April 15, 2013 and is computed based on the Cumulative Match Report performed by the data analysis group in this study [36]. The simulated kidney exchange pools have a number of donor-patient pairs varying from 30 to 200 nodes and the percentage of altruistic donors is chosen according to the value reported by Saidman generator.

For each experimental run, the compatibility matching rules encoded in the simulated datasets differ according to the random generation of a kidney pool. However, it is likely that the larger pool size results in existing more compatible pairs. Table 2 illustrates the compatibility information with respect to various pool sizes. To satisfy the real life hospitals constraints, we set the maximum length for cycles and chains to three, k = 3. The total number of extracted chains and cycles is also shown in Table 2.

**Table 2:** Compatibility information with respect to the pool size, n.

	Compatibility Information				
	# Arcs	# Chain(2)	# Chain(3)	# Cycle(2)	# Cycle(3)
n = 30	56	1	3	2	1
n = 40	108	13	26	3	3
n = 50	123	3	11	7	9
n = 75	238	15	38	3	8
n = 100	482	15	79	10	25
n = 200	1705	53	363	21	170

As shown in Table 2: for the KPD pool that contains 200 nodes, there are 1705 compatible relations (arcs) between nodes. Also, it contains 53 chains of length two and 363 chains of length three, while the number of cycles of lengths two and three are 21 and 170 respectively. On the other hand, KPD pool of size 30 contains 56 compatible relations. Also, the pool contains a single chain of length two and 3 chains of length three and the number of cycles of lengths two and three equals 2 and 1 respectively. It is noted that when the pool size increases, the total number of compatible relations increases. As a result, the total number of extracted chains and cycles consequently increases. The extracted chains and cycles together compose all of the available exchanges in KPD pool.

Once chains and cycles are extracted, the optimization step is initiated to select the optimal number of feasible exchanges, which in turn maximizes the KPD utility. We compared our proposed method to Genetic Algorithm(GA), which is the only known stochastic-based optimization algorithm applied to the kidney exchange space [4, 12]. GA was utilized to search for the maximum number of feasible exchanges in the extracted chains and cycles using the fitness function defined by Eq (18). The parameters setting for the applied optimization algorithms is listed in Table 3. Crossover and mutation probabilities for GA were set to 0.8 and 0.2 respectively redand the used selection method was Roulette Wheel. For simplicity, this method is mentioned as GA-KPD.

**Table 3:** Parameter settings for GA-KPD and the proposed method.

Parameters	GA-KPD	Proposed
Max number of iterations (n)	100	200
Population size	800	200
Individual Dimension	# number of cycles + # number of chains	
Search Domain	[0 1]	

**Fig 8:**
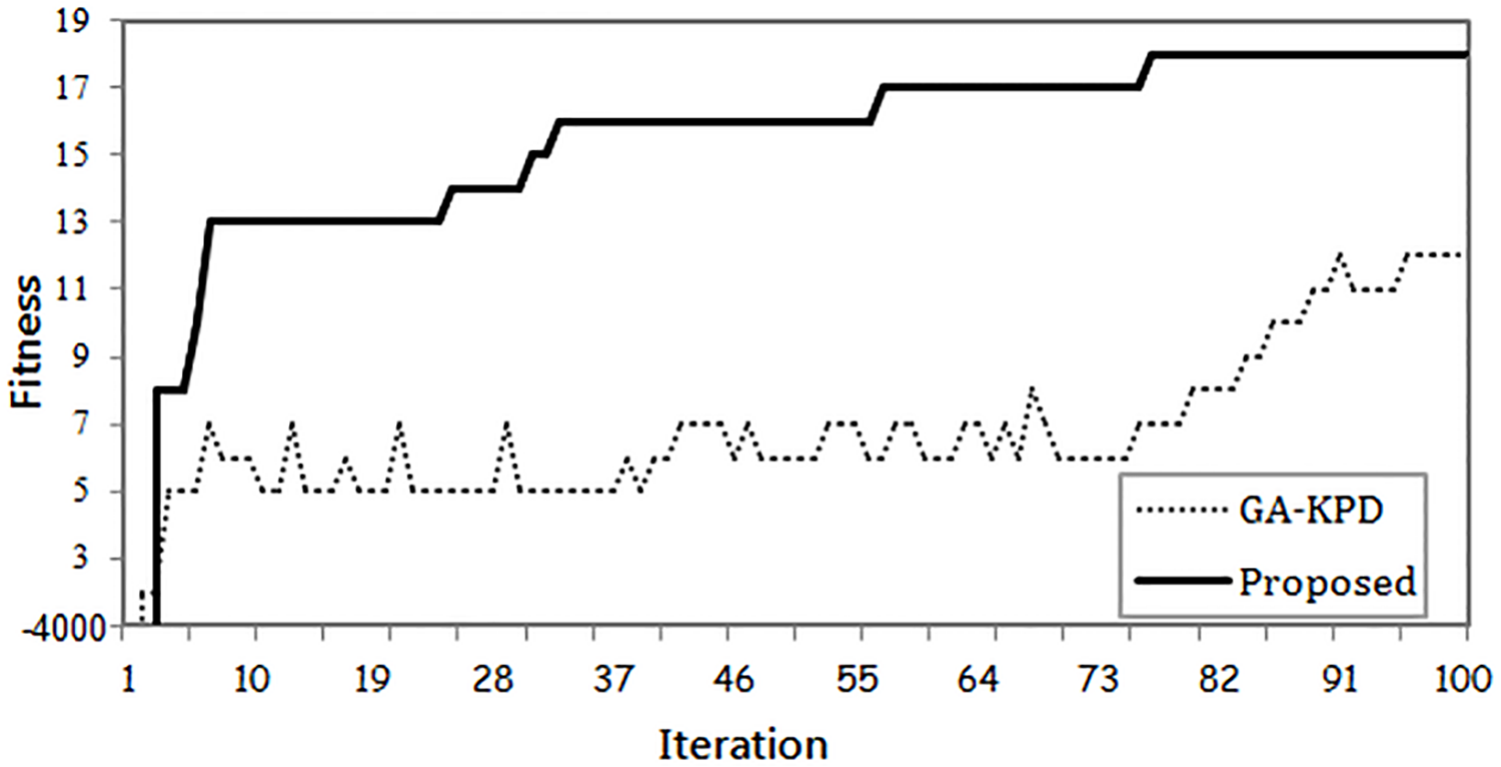
Elite individual’s fitness per iteration.

To compare the performance of the applied optimization methods, Ten empirical experiments were performed for the same KPD pool. Each experiment is initiated with a randomly generated population and the average results are reported. Fig 8 shows the convergence curves of the proposed method compared to GA-KPD. For each experiment, the fitness value of the elite individual is recorded per iteration.

As illustrated in Fig 8, the adopted Ant Lion Optimization algorithm in our proposed method has the fastest convergence capability towards the optimal solution. The proposed method needs less than 90 generations to converge. On the other hand, GA-KPD could not converge to the optimal solution for the maximum number of generations equals 100.

Also, we compared our results with a deterministic-based algorithm for kidney exchanges, Integer Programming (IP), which is utilized for finding the optimal number of exchanges in the extracted chains and cycles. We will call this method IP-KPD and its problem formulation is defined by the following equation:

$$\begin{array}{c} \begin{array}{cc} \text{maximize} & \sum_{\text{c}\in \text{M}}^{}\sum^{\text{U}}{\text{x}}_{\text{c}}{\text{w}}_{\text{i},\text{j}}\\ & \text{s}.\text{t}.\;{\text{x}}_{\text{c}}\in \left\{0,1\right\},\forall \text{c}\in \text{M}\\ & \;\sum_{\text{c}\in \text{M}(\text{t})}{\text{x}}_{\text{c}}\leq 1,\forall \text{t}\in \text{Pool} \end{array} \end{array}$$ (20)

where M is the set of all extracted chains and cycles from KPD pool up to the maximum length equals three. M(t) is a chain or cycle in M that contains node t, which represents an altruistic or a donor-patient pair. w_i,j_ denotes a utility value for the compatible relation between donor i and patient j, and *x*_*c*_ is a binary vector representing whether the chain or cycle c is selected for transplant (x_c_ = 1) or not (x_c_ = 0). The existing constraints are stated as no node can be used more than one in a solution.

**Fig 9:**
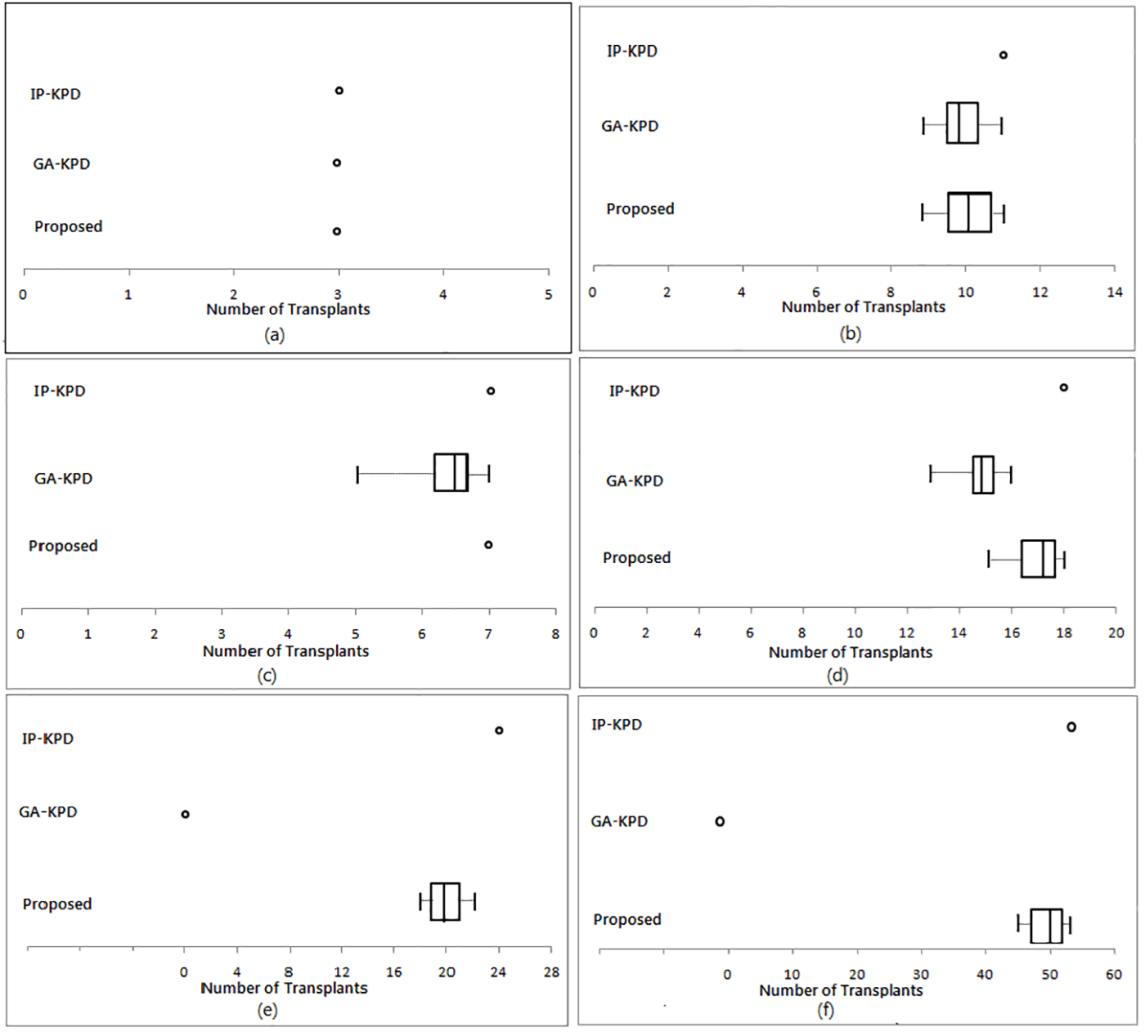
Transplants returned by all applied methods. (a) Pool size = 30, (b) Pool size = 40, (c) Pool size = 50, (d) Pool size = 75, (e) Pool size = 100, (f) Pool size = 200.

To compare the performance of all methods, another set of experiments is executed. All experiments are tested for different pool sizes. Experiments for GA-KPD and the proposed method are performed using the parameters specified in Table 3. Also, all experiments are repeated for ten times to account for possible statistical variations. Fig 9 shows experiments results in terms of the number of exchanges (transplants) produced by all applied methods for KPD pool sizes equal 30, 40, 50, 75, 100, and 200. The best, average, and worst number of resulting transplants are illustrated in the graph.

We can observe that when the pool size was 30, all applied methods gave the same number of transplants. When pool size is greater than 50, GA-KPD fails to converge and produce infeasible solutions, i.e. the number of resulting transplants are considered zeros. Also, we can observe that the proposed method performs better than GA-KPD. The searching strategy of ALO is more efficient than GA due to the enhanced exploration, exploitation, and convergence capabilities of ALO over GA. Moreover, it is clear that the proposed method succeeded to provide a very close performance to IP-KPD in terms of the number of resulting transplants. Table 4 shows the average number of transplants/ exchanges with the standard deviation, returned by GA-KPD and the proposed method, compared to the exact number of exchanges returned by IP-KPD.

**Table 4:** Number of transplants produced by methods(GA-KPD, Proposed, IP-KPD) for various KPD pool sizes. M denotes total number of extracted exchanges from KPD pool.

Pool Size	M	# Transplants		
		GA-KPD	Proposed	IP-KPD
n = 30	7	3 ± 0	3 ± 0	3
n = 40	45	9.8 ± 0.8	10.2 ± 0.98	11
n = 50	30	6.6 ± 0.7	7 ± 0	7
n = 75	64	15.4 ± 0.98	17.4 ± 0.92	18
n = 100	129	*	19.8 ± 1.5	24
n = 200	607	*	50.2 ± 3.16	53

From Table 4, we can observe that, the number of extracted exchanges (chains/ cycles) does not imply anything about the number of resulting transplants. For example, when the pool size equals 100, the total number of exchanges was 129 and the optimal number of transplants produced by IP-KPD was 24. Also, we can see that our proposed method generally provides an average number of transplants greater than GA-KPD. When the number of exchanges was greater than 64, GA-KPD fails to provide any feasible solution (indicated in the table by ‘*’). Additionally, the maximum number of transplants produced by our proposed method is very close to IP-KPD in almost all cases.

**Fig 10:**
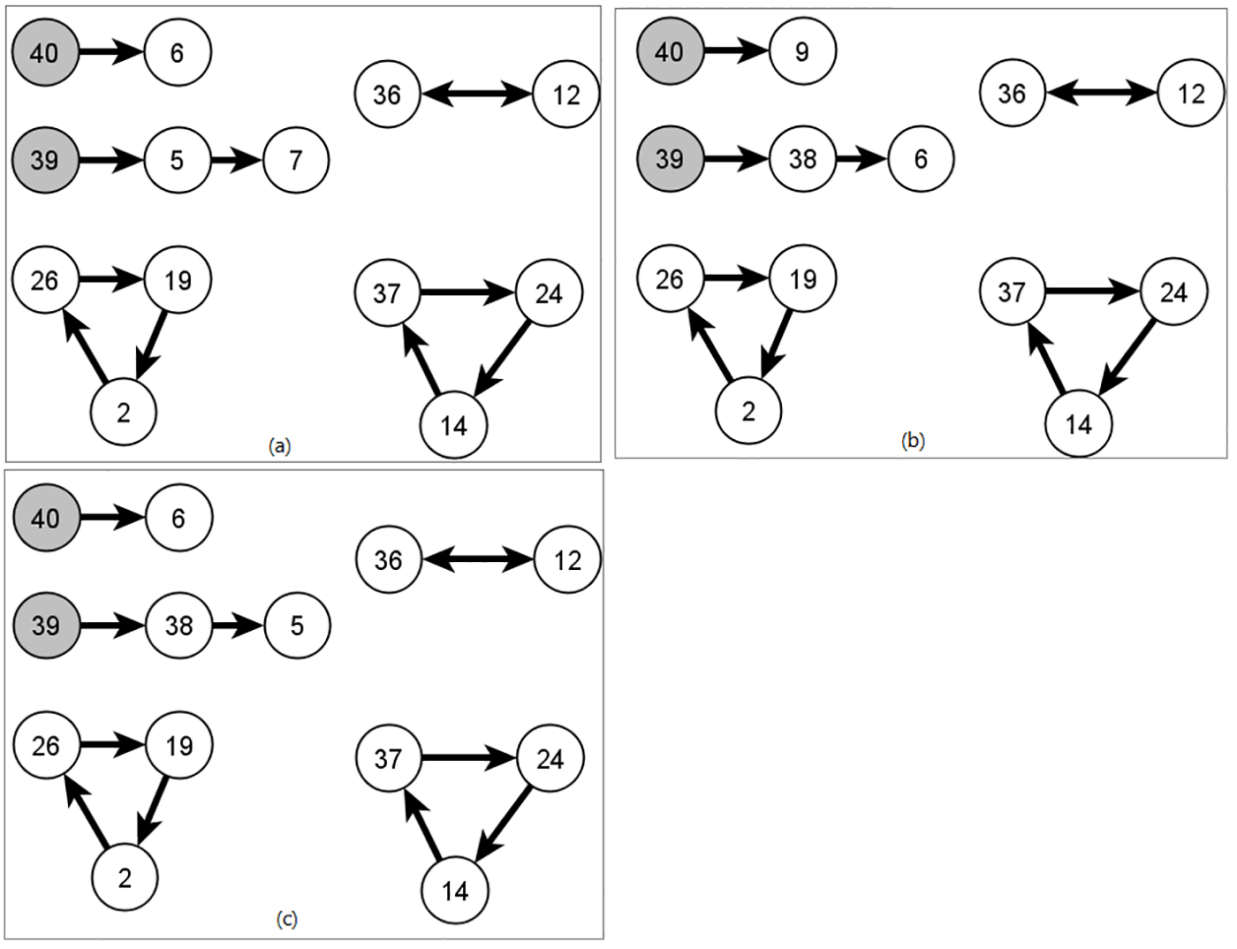
The composition of a resulting optimal solution. (a) IP-KPD, (b) Proposed, (c) GA-KPD.

Moreover, we compared the optimal solutions generated by all methods, Fig 10 depicts an example for the composition of a generated solution for the pool size equaling 40. The chains and cycles of the solutions produced by IP-KPD, proposed method, and GA-KPD are shown in Fig 10(a), 10(b) and 10(c), respectively. In all figures, the altruistic node is visualized as a gray-filled circle where the non-filled circles indicate the nodes of patient-donor pairs.

The compatibility relations varies among the pool pairs and consequently the compared methods can generate different solutions with the same number of transplants. In Fig 10, the optimal solution produces 11 transplants and contains two chains with lengths two and three, and three cycles one of length two and the others of length three.

The computational running time of the produced solution is also important because patients can not wait too long for a kidney transplantation. Therefore, another important goal for our applied optimization algorithm is providing matches to patients as soon as possible. Table 5 lists the computational running time for all applied methods. Recall that the time reported for GA-KPD and our proposed method is the average running time for ten different runs. Matlab is used for implementing all of the utilized methods, all experiments ran on Windows 8 machine with Core i7 2.7-GHz Intel processors and 4-GB RAM.

**Table 5:** Elapsed running time (in seconds) for all methods in various KPD pool sizes. * indicates runs giving infeasible solutions.

Pool Size	GA-KPD	Proposed	IP-KPD
n = 30	13.8	**1.89**	0.04
n = 40	24.6	**3**	0.07
n = 50	22.4	**2.62**	0.05
n = 75	29.3	**3.87**	0.18
n = 100	68.5*	**4.57**	0.47
n = 200	1290.5*	**14.2**	7.1

It is remarkable to mention that the Ant Lion optimization algorithm converges faster than GA, which needs larger population size and additional computational steps accordingly. Despite this, GA fails to converge for large pool sizes and gives infeasible solutions. Moreover, our proposed method for kidney exchanges overcomes the computational complexity of the one proposed in [4]. In our KPD problem formulation, the process of extracting all cycles and chains from a kidney exchange pool is executed only once, which reduces the overall computational running time.

Moreover, In order to study the effect of the introduced post-processing step on the kidney exchange results, we compared our results with/without including it. Table 6 lists the max, average, and min numbers of returned transplants for each case.

**Table 6:** Number of transplants (with) and without the proposed post-processing step for various KPD pool sizes.

Pool Size	Max	Average	Min
n = 30	(3)3	(3)3	(3)3
n = 40	(11)11	(10.2)10.2	(9)9
n = 50	(7)7	(7)7	(7)7
n = 75	(18)18	(17.4)17.4	(15)15
n = 100	(22)21	(19.8)19.4	(18)18
n = 200	(53)49	(50.2)45	(45)41

For a pool size less than 100 nodes, we can observe that the post-processing step has no effect on the final solutions (i.e. ALO produces the global optimal solutions). On the other hand, for large KPD pool sizes, some ALO runs trapped in the local optimal regions of the search space. Consequently, the post-processing step plays a key role to improve the non optimal solutions given by ALO for large pool sizes.

The most important advantage of our proposed method is the flexibility of its application in the dynamic kidney exchange environments. Recall that the first term (subgoal) of the proposed fitness function defined in Eq (18), denotes the number of given transplants (if w_i,j_ all equals 1). By assigning different utility values for each compatible relation w_i,j_, our method can be adopted easily to handle on-line exchanges. For example, a high utility value can prioritize the hard-to-match patients among other pool pairs and increasing their possibility for selection by the optimization algorithm. Additionally, other factors such as patient’s registration time, patient’s departure time, etc. could be easily included in the ALO algorithm fitness function in order to take decisions based on the multi-criteria optimized solutions for kidney exchanges.

## 4 Conclusion

Motivated by the increasing number of terminal stage renal patients who have the only option of kidney transplantation for saving their lives, kidney paired donation (KPD) programs are recently introduced for increasing the number of kidney exchanges on a large scale. The dynamic nature of KPD problem and the large size of its candidates’ pool complicate the process of searching for the optimal number of possible exchanges. In this paper, we formulate the KPD as a combinatorial optimization problem using stochastic-based Ant Lion Optimization algorithm. The proposed novel method searches efficiently for the optimal number of exchange cycles and chains in a given KPD pool considering the constraints imposed by the limited computational and operational hospital resources. The stochastic-based ALO algorithm opens the future of taking fast and customized-based decisions for kidney exchanges considering the dynamic nature of the problem and its multi-criteria optimized solutions. Our proposed method has comparable exchange results to other competing tools including deterministic IP, and outperforms stochastic GA in terms of its speed and the quantity of resulted optimal solutions. One of our future insights is updating the current implementation of ALO algorithm-based program to include multi-criteria objective function and make it more informative and usable for patients and hospitals. Ant Lion Optimization algorithm-based program will bring more hope for caring patients with terminal-stage renal diseases and adjusting the expectancy of their life quality by taking the optimal decisions at the right time.
